# Associations of Physical Activity and Sedentary Time in Primary School Children with Their Parental Behaviors and Supports

**DOI:** 10.3390/ijerph15091995

**Published:** 2018-09-13

**Authors:** Chiaki Tanaka, Masayuki Okuda, Maki Tanaka, Shigeru Inoue, Shigeho Tanaka

**Affiliations:** 1 Division of Integrated Sciences, J. F. Oberlin University, Tokyo 194-0294, Japan; 2 Department of Environmental Medicine, Graduate School of Science and Engineering, Yamaguchi University, Yamaguchi 755-8505, Japan; okuda@yamaguchi-u.ac.jp; 3 Department of Early Childhood Education, Kyoto Bunkyo Junior College, Kyoto 611-0041, Japan; m-tanaka@po.kbu.ac.jp; 4 Department of Preventive Medicine and Public Health, Tokyo Medical University, Tokyo 160-8402, Japan; inoue@tokyo-med.ac.jp; 5 Department of Nutrition and Metabolism, National Institute of Health and Nutrition, National Institutes of Biomedical Innovation, Health and Nutrition, Tokyo 162-8636, Japan; tanakas@nibiohn.go.jp

**Keywords:** exercise, moderate-to-vigorous physical activity, parental supports, sports, accelerometer

## Abstract

Background: The associations of objectively evaluated moderate-to-vigorous physical activity (MVPA) and sedentary time between primary school children and their fathers or mothers have not been fully understood. Therefore, we tested the associations in children. *Methods*: The participants were first to sixth grade boys (n = 166, 9.4 ± 1.6 years) and girls (n = 202, 9.4 ± 1.6 years) and their parents (fathers, n = 123 and mothers, n = 321). MVPA and sedentary time were measured using triaxial accelerometry. The relationship between parental support which was assessed by self-reported questionnaire and children’s MVPA was also examined. Results: MVPA in the children was positively correlated with maternal MVPA after adjustment for the children’s gender, grade, body mass index z-score, paternal or maternal age, and school (*p* < 0.001). However, paternal or maternal sedentary time and paternal MVPA showed no significant association with sedentary time or MVPA in children. On the other hand, the percentage of MVPA in children who spent more time with their mothers on weekends was significantly lower than those who spent less time (*p* = 0.034). Children whose mothers watched their sports events had a significantly higher percentage of MVPA than those whose mothers did not watch these events (*p* = 0.008). There were no associations between children’s MVPA and paternal support. Conclusions: The findings of this study demonstrate the significance of maternal MVPA and support.

## 1. Introduction

Sufficient physical activity (PA) and a low level of sedentary behavior are important for health in school-aged children and youth [1,2,3]. A previous systematic review reported longitudinal changes in objectively evaluated total sedentary time in school-aged children and youth [4]. The weighted mean increase in daily sedentary time per year was 5.7% for boys and 5.8% for girls, equating to approximately 30 min of extra daily sedentary time during the year. Recently, Jago et al. [5] reported the changes that occurred in PA and sedentary time of children from the Bristol (UK) B-PROACT1V cohort aged between 5–6 and 8–9 years. The results showed there were similar increases in sedentary time in these girls and boys, with the decrease in moderate-to-vigorous PA (MVPA) being more marked in girls [5]. It is therefore desirable to offset these decreases in MVPA and increase in sedentary time by promoting PA and reducing sedentary time in the early years of childhood.

It has been suggested that parents play a critical role in developing and shaping the PA and sedentary behavior of their children through role modelling [6,7,8]. Previous studies on primary school aged children and their parents have examined the associations between objectively measured PA or sedentary time in parents and their children [6,7,9]. However, a recent systematic review pointed out that only a few studies have examined the effect of fathers on children’s PA and this relationship remains unclear [10]. A recent longitudinal study in the UK found that there was little evidence that either male or female parent MVPA at 5–6 years of age predicted child MVPA at 8–9 years of age. Similar associations were observed for sedentary time. There is also little evidence that changes in parent MVPA or sedentary time predict changes in MVPA or sedentary time in children, respectively. It is suggested that interventions that aim to increase the activity levels of children by increasing their parent’s levels are unlikely to have a marked impact on improving childhood activity at a population level [11]. In this regard, it is unlikely simple strategies that focus on encouraging parents to be active together with their child will be sufficient to increase PA in children [12].

Previous studies have shown that parents can influence the health behavior of their children by engaging in supportive behavior [13,14,15,16,17]. For example, greater parental support for PA was reported to be associated with greater objectively evaluated child PA in UK and USA studies [13,14,15,16,17]. However, the contents of the parental support activities were not consistent between these studies. Moreover, these previous studies analyzed two sub-factors: logistic support and explicit modeling. The relationship between parental support for PA in primary school children and the children’s PA should therefore be examined in greater detail. Especially, no previous studies examined the relationships between both mother’s and father’s support and child’s PA. In these previous studies questions on parental support were addressed to only one parent, and the majority was mothers’ answers. Thus, the potentially important role of different sex parental support should be examined. Moreover, an association between parental support and sedentary time in children is little known.

The purpose of this study was to examine the associations between objectively evaluated sedentary time and MVPA in primary school children and their fathers or mothers. The relationship between fathers’ or mothers’ supports and children’s MVPA or sedentary time was also examined.

## 2. Materials and Methods

The study was a cross-sectional design that investigated Japanese children and their parents. A total of 166 boys (their 148 mothers and 58 fathers) and 202 girls (their 173 mothers and 65 fathers) from 14 primary schools in urban areas of Tokyo and Kyoto were included in the convenience sample. Flyers were distributed in the schools to invite participants to join the study and all participants and their parents gave their informed consent. The study protocol (receipt number, 12,023) was approved by the Ethical Committee of J. F. Oberlin University. Data for anthropometric measurements, sedentary time, and PA were collected during the school year between June 2012 and January 2015 (June, September, October, and November 2012, on April, October, and November 2013, on November 2014, and on January 2015).

### 2.1. Objective Measurement of Sedentary Time and Physical Activity

A triaxial accelerometer (Active Style Pro HJA-350IT, Omron Healthcare, Kyoto, Japan) with dimensions of 74 × 46 × 34 mm and weight 60 g including batteries was used to measure sedentary time and PA. The participants wore the accelerometer on the left side of the waist during the school year. The details of the accelerometer are described elsewhere [18]. Briefly, the synthetic acceleration of three axes was calculated using signals before and after high-pass filtering using 10-s Epoch to remove the gravitational acceleration component from the signal. The ratio of unfiltered to filtered acceleration was then calculated to classify the type of PA: ambulatory activities such as walking and running and non-ambulatory activities such as playing games, playing with blocks, tossing a ball, cleaning and clearing away. The accelerometer provides metabolic equivalents (METs) [19]. For parents, the METs provided by the accelerometer were used. However, the METs are overestimated for primary school children because the predictive equations used for the Active style Pro accelerometer were established for adults [20]. Therefore, the following conversion equations for primary school children obtained from the results of Hikihara et al. [20] were used: Ambulatory activities, 0.6237 × MET value from the Active style Pro + 0.2411; non-ambulatory activities, 0.6145 × MET value from the Active style Pro + 0.5573.

Sedentary time and MVPA were monitored continuously for 7 days. The participants were requested to wear the accelerometers at all times, except under special circumstances, such as dressing, bathing, or swimming. We analyzed the accelerometer data of children collected between 7:00 and 21:00 to exclude sleep time and to distinguish sedentary time from sleep time. We also excluded sleep time from the accelerometer data of parents using the parents’ log sheet. We included days in which the device had been worn for more than 600 min (10 h), not including the time elapsed for the above stated unavoidable reasons [21]. Our analysis included participants with data available from at least 2 weekdays and 1 weekend day.

### 2.2. Anthropometric Measurements

For the anthropometric measurement, body height and weight were measured without shoes, but with clothing. We measured some children’s clothes, and the average weight was 0.7 kg. The weight of clothing (0.7 kg) was subtracted from the measured body weight to calculate net body weight on the first day that the students were given accelerometers. This net body weight was then used to calculate body mass index (BMI, kg/m^2^). The chronological age was calculated as the difference between the birthdate and the first date of accelerometer assessment [22,23], while the students’ calendar age was calculated accurately from the month and year of birth until the first day of measurement.

### 2.3. Self-Reported Measures

Questionnaire data were collected from the parents with their parental behavior evaluated by the following six questions. Q1, “How much time do you spend with your child on weekdays?”—0, 30 min, 1, 2, 3, 4, or >5 h; Q2, “How much time do you spend with your child on weekends?”—0, 30 min, 1, 2, 3, 4, or >5 h; Q3, “How often do you encourage your child to be physically active?—rarely, occasionally, often, or always; Q4, “Do you watch your child’s sporting events?”—rarely, occasionally, often, or always; Q5, “Do you think that it is important for you to pay attention to sports?”—unimportant, somewhat important, important, or very important; and Q6, “Does your family enjoy sports or physical activities as a family recreational activity?”—rarely, occasionally, often, or always. We also asked parents’ occupation, work pattern (full-time work, part-time work) and jobs on Saturday and Sunday. Parents who had jobs on Saturday and Sunday were considered as having weekend job duty.

### 2.4. Statistical Analysis

The sedentary time and MVPA for each time period were calculated using METs, in which the average number of weekday or weekend minutes spent in sedentary time (≤1.5 METs) and MVPA (≥3.0 METs) was calculated for each individual. The average and percentage weekly values were then calculated using weighting for 5 weekdays and 2 weekend days (weighted data = (Average for 2 weekdays × 5 + average for 1 weekend day × 2) ÷ 7). PA assessed by the accelerometer was expressed as PA status for ambulatory activity or non-ambulatory activity in MVPA. 

**Figure 1:**
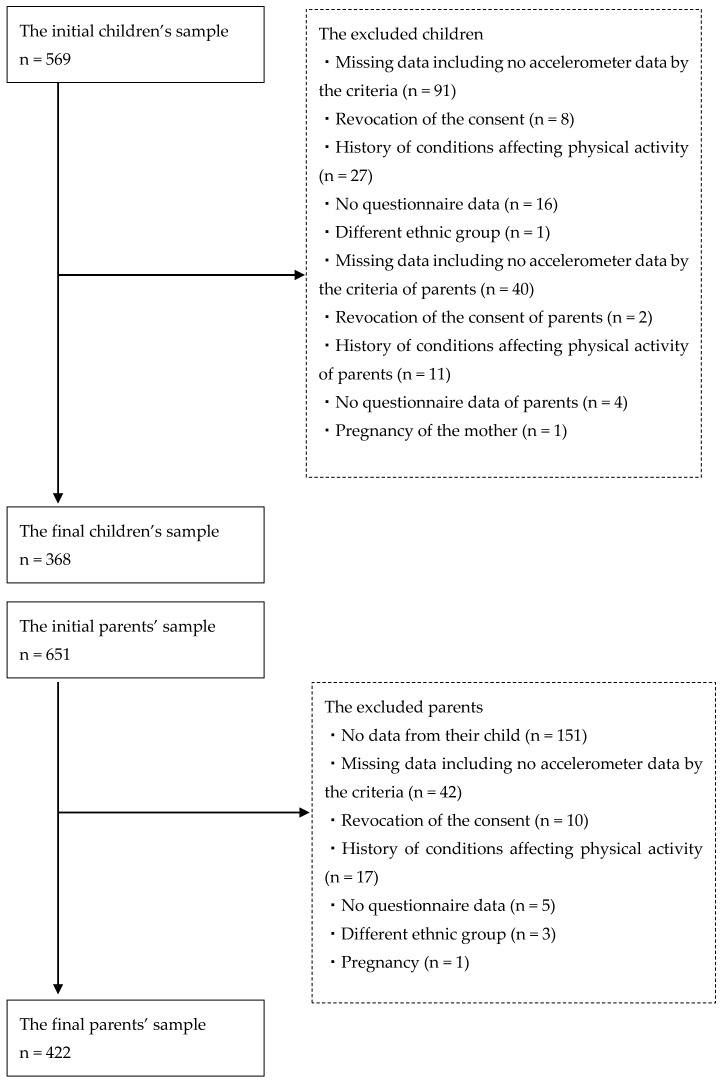
Flow of participants.

In the questionnaire, responses to Q1 and Q2 were categorized into two groups according to the distribution of each answer. Q3, Q4, and Q6 were categorized into “yes” or “no” according to the answers of “rarely and occasionally” or “often and always”. Q5 was categorized into “unimportant”, “somewhat important” or “important” according to the answers of “unimportant”, “somewhat important” or “important and very important”. The associations between children’s percentage of MVPA (%MVPA) or % sedentary time and their parent’s activity or behavior were examined using mixed linear regression models. MVPA and sedentary time were divided by total wear time to obtain %MVPA and %sedentary time, respectively. We analyzed the associations between factors of the children’s mothers and fathers, separately. Gender, grade, and body mass index z-score of the children were included in the models as confounders. Families nested in the schools were also included as random effects. When the parent’s activity was an independent variable, their age was included as a cofounder. As summarized in Figure 1, the initial children’s sample comprised 569 participants. Due to missing data including no accelerometer data by the above-mentioned criteria (n = 91), revocation of the agreement (n = 8), history of conditions affecting PA, such as respiratory disease or heart disease (n = 27), no questionnaire data (n = 16), different ethnic group (n = 1), missing data including no accelerometer data by the criteria of parents (n = 40), revocation of the consent of parents (n = 2), history of conditions affecting PA of parents (n = 11), no questionnaire data of parents (n = 4), and pregnancy of the mother (n = 1), the final study sample comprised data from 368 children. The initial parents’ sample comprised 651 participants. Due to missing data including no accelerometer data, no data from their child (n = 151), no above-mentioned criteria (n = 42), revocation of the agreement (n = 10), history of conditions affecting PA, such as respiratory disease or heart disease (n = 17), no questionnaire data (n = 5), different ethnic group (n = 3), and pregnancy (n = 1), the final study sample comprised data from 422 parents. There was no significant difference in the age, body weight, and body height of the study group and the children who withdrew from the study. There was no significant difference in the age of the study group and the parents who withdrew from the study. The statistical analyses were performed using SAS 9.4 (SAS Institute Inc., Cary, NC, USA), with *p* < 0.05 considered statistically significant.

## 3. Results

### 3.1. Characteristics of the Study Participants

The mean age of the mothers of the 148 boys was 41.0 ± 4.2 years and 42.5 ± 4.9 years for the fathers of the 58 boys, while the mean age of the mothers of the 173 girls was 41.3 ± 3.9 years and 43.6 ± 4.7 years for the fathers of the 65 girls. The characteristics of the children are summarized in Table 1. Of the study participants, 13.3% of boys and 5.4% of girls were overweight/obese, while 2.4% of boys and 5.4% of girls were classified as thin. Times in ambulatory and non-ambulatory MVPA, and sedentary time were 2.7% (SD 1.5%), 3.4% (SD 2.1%), and 55.5% (SD 8.8%) for mothers, and 4.3% (SD 2.4), 1.8% (SD 1.6%), and 64.9% (SD 11.1%) for father, respectively. Out of 146 children (47.3% of all children for parental support analysis) who answered their mother’s work pattern by the questionnaires, 41 children had mothers with full-time work. The number of children who answered their father’s work pattern by the questionnaires were 71 (51.4%). All of their fathers worked full-time. One hundred and fifty-six children’s mothers (51.0%) and 111 children’s fathers (80.4%) answered weekend job duty. One hundred and one children’s mothers and 69 children’s father had no duty on weekends or no job. Mothers without a job were 117 from 287 (93.8%) children who reported mother’s occupation. Table 2 shows the results of parental support. There were no gender interactions in children. Objectively evaluated MVPA in children showed a significant positive association with maternal total MVPA (*p* < 0.001) and ambulatory MVPA (*p* = 0.001) after adjustment for the children’s gender, grade, body mass index z-score, paternal or maternal age, and school (Table 3). Non-ambulatory MVPA in children showed a non-significant positive association with maternal non-ambulatory MVPA (*p* = 0.062) (Table 3). On weekdays, objectively evaluated MVPA in children also correlated positively with maternal total MVPA (*p* < 0.001) and ambulatory MVPA (*p* = 0.007). On weekend days, objectively evaluated ambulatory MVPA in children correlated positively with maternal ambulatory MVPA (*p* = 0.030). Paternal or maternal sedentary time and paternal MVPA showed no association with either sedentary time or MVPA in children.

### 3.2. Associations of Objectively Evaluated Physical Activity and Sedentary Time between Primary School Children and Their Fathers or Mothers, and Parental Supports

The %MVPA in children who spent more time with their mothers on weekends was significantly lower than that of children who spent less time with their mothers (difference between groups = −0.901; *p* = 0.034) (Table 4). When limited to full-time working mothers or mothers without a job, there were no significant differences in %MVPA of children (n = 37: difference between groups = −0.004; *p* = 0.463, n = 110: difference between groups = −1.051; *p* = 0.111, respectively). Children whose mothers watched their children’s sports events had a significantly higher %MVPA than those whose mothers did not watch these events (difference between groups = 0.720; *p* = 0.009). There were no significant differences in %MVPA of children, when limited to full-time working mothers (difference between high and low groups = 0.398; *p* = 0.640) or mothers without a job (effect size = 0.532; *p* = 0.209). On the other hand, as shown in Table 5 children who spent time with their mothers on weekends had a significantly longer %sedentary time than that of children who spent less time with their mothers (47.0% vs. 44.4%, difference between groups = 2.540; *p* = 0.037). The difference increased when limited to full-time working mothers (difference between groups = 2.5898, *p* = 0.740) or mothers without a job (difference between groups = 4.698; *p* = 0.031). There were no significant associations between MVPA or sedentary time of the children and paternal support. There were no significant associations between MVPA of the children who spent more time with their fathers who had the day off on weekends (n = 69, difference between groups = 0.004, *p* = 0.513). Due to the small sample size, further detailed analyses were not performed for the other subgroups.

## 4. Discussion

The findings in this paper demonstrate that only a mother’s MVPA was associated positively with their child’s MVPA. The mother’s sedentary time was not associated either positively or negatively with their child’s sedentary time. This study also found positive associations between children’s MVPA and maternal support. Conversely, children whose mothers spent time with them on weekends had a lower MVPA and higher sedentary time. There were no associations between children’s MVPA or sedentary time and either paternal MVPA, sedentary time, or support.

The present study collected data between June 2012 and January 2015. In our previous study, significant seasonal variation in MVPA and sedentary time was found between spring season in the school year and summer season in the summer vacation [24]. However, there is no study of seasonal variation in PA and sedentary time among winter and spring or autumn seasons in Japan. The mean values of maximum temperatures in the present study were 18.0 (SD 2.4) degree in the spring season (April), 18.8 (SD 2.1) degree autumn season (from September to November), and 12.9 (SD 4.2) degree in the winter season (January) [25]. All measurements were conducted under moderate weather, without heavy rain or snow, storm, etc. Recently, Lewis et al. reported that daily maximum temperature was significantly associated with MVPA time in Australia and Canada [26]. MVPA time appears to be optimal when the maximum temperature ranges between 20 and 25 °C in both countries. Thus, the slight difference between seasons in 4 years would not largely affect the results of the present study. Moreover, measurements were performed in January for only 21 children and their parents. In addition, because effect of school has been statistically adjusted and measurements were performed in a few weeks in each school, the effect of season on the results would be minimum.

The findings of our study demonstrated no associations between paternal MVPA and sedentary time, or the sedentary time of mothers and their children. In contrast, a mother’s MVPA showed a positive association with their child’s MVPA. Previous studies have shown significant relationships between the activities of parents and their children, including both sedentary and MVPA [6,9,11,27,28]. However, not all these studies are in agreement [29]. Jago et al. [29] also reported there was a weak, but significant association between sedentary time in parents and children. In terms of parental gender, maternal and paternal sedentary time showed similar associations with child sedentary time [11], while the association with child MVPA was lower in fathers [11]. Fuemmeler et al. [6] also reported that the minutes of MVPA and sedentary time in mothers and fathers correlated positively with both child MVPA and sedentary time. These previous studies were in UK and USA [6,9,11,27,28,29]. Hallal et al. reported that physical inactivity in adults (15 years or older) in UK or Japan were higher than that of in USA [30]. The proportion of 13–15-year-old boys and girls not achieving 60 min per day of MVPA is similar both UK and USA, whereas there are no such data in Japan [30]. Thus, the difference in findings between these studies may be related to different PA conditions due to the different locations (Western countries vs. Japan). The sample sizes in previous studies were from 45 parents to 408, and the majority of parental participation were mothers. Compared to fathers, mothers seem to spend more time in non-ambulatory activities such as household work (e.g., dusting, vacuuming, dishwashing, and doing laundry) [31]. In addition, the NHK National Time Use Survey [32] reported that over 90% of Japanese women carry out housework on weekdays and weekends, whereas only 41% of men do such work on weekdays, 51% on Saturdays, and 56% on Sundays. As a result, women spend about 4 h 30 min on housework each day, while men spend about 50 min on weekdays, 1 h 23 min on Saturdays, and 1 h 33 min on Sundays. These data demonstrate that Japanese mothers may spend more time than Japanese fathers carrying out activities classified as either household or child-care. The present study showed maternal non-ambulatory activities (3.4%) were higher than ambulatory activities (2.7%), which was consistent with the previous study [31] with the opposite association being observed in fathers (non-ambulatory activities: 1.8% and ambulatory activities: 4.3%). Gender differences in lifestyle characteristics may therefore affect maternal and paternal PA and sedentary time, which in turn may lead to an association with their children’s PA and sedentary time.

A recent longitudinal study in the UK found that parents who were more physically active when aged 8–9 years had relatively active children, although the magnitude of this association was generally small. Furthermore, changes in the PA of parents was found to be a weak predictor of changes in the PA of their child [11]. Simple strategies that focus on encouraging parents to be active together with their child are therefore unlikely to be sufficient to increase a child’s PA [12]. Our data provides no evidence of an association between fathers’ support and their child’s MVPA or sedentary time although there were positive associations between maternal support of a child’s sporting events and the child’s MVPA. We showed that 44% of fathers spent less than 1 h with their child on weekdays, and even on weekends, only 35% of fathers spent more than 5 h with their child. During weekdays, in particular, fathers spent little or no time with their child. According to the NHK National Time Use Survey [32], the working hours of Japanese male jobholders in their 30s and 40s was more than 9 h on weekdays. Although 40% of mothers spent more than 5 h with their child on weekdays and 89% did so on weekends, the children’s objectively evaluated MVPA during the weekend showed a negative association with the length of time the mothers spent with their child. On the other hand, the children’s objectively evaluated sedentary time correlated positively with the sedentary time for mothers. As mentioned above, considering that over 90% of Japanese women carry out housework on weekdays and weekends and spend about 4 h, 30 min on housework each day according to the NHK National Time Use Survey [32], even if the mother and child spend time together, they might engage in more sedentary behavior than in active PA. The present study did not directly evaluate joint PA between parents and their children as we wanted to reduce parents’ burden as participants. Dunton et al. [33] examined the locations of joint PA between parents and children using accelerometers and global positioning systems (GPS) devices. They found joint PA was spread across residential locations (35%), commercial venues (24%), and open spaces and parks (20%). Because a mother’s objectively measured MVPA was associated positively with their child’s MVPA, future studies should examine the activities that mothers and their child carry out together.

As shown in Table 4, the only positive associations we observed were between maternal support of children’s sporting events and the MVPA of children, despite the parental support of children’s sporting events being similar between parents (mothers 55.9% vs. fathers 48.1%). It might be a positive aspect to improve the MVPA of children. To our knowledge, only four studies in the US and UK [13,14,15,16] have examined the role of family factors in promoting PA and used the Activity-Related Parenting Practices Scale as a measure of parental support [34]. This scale consists of a seven-item scale with two sub-factors: logistic support (attending the activities with the child, three items) and explicit modeling (using PA as a family recreation, four items). Previous studies showed parental logistical support [14,15,16] or explicit modeling [13] was associated with the MVPA of their children. Forthofer et al. [17] also reported that mothers’ support (4 items, encourage PA, play outside and do PA, provide transportation, and watch child be physically active) had modest positive associations with MVPA. The findings of the present study were consistent with some of these results, despite our study treating each of the items as a single factor. We also examined associations of MVPA and sedentary time between children and parental supports with parents’ work pattern and jobs on Saturday or Sunday. However, there were no significant differences of %MVPA and %sedentary time, maybe due to the small sample size. Although the previous studies didn’t report parental occupations, the different association between father or mother and their children may be mediated by parents’ work pattern. It is also possible that the different findings we have described may actually be due to the different locations of the studies (UK vs. Japan), because a comparison study of leisure time and personal care time in full-time worker among OECD, those times were shortest in Japan [35]. In this regard, simple strategies that focus on parental support may not be sufficient to increase PA in children. Therefore, the importance of parental support should be emphasized in policy and intervention development for families with a non-full-time worker. Rather, we would argue that more work is needed to identify whether or not children with full-time worker parents have low PA.

Previous studies also reported that parenting style was associated with children’s PA [14,15,16]. For example, in a computer-assisted telephone interview survey in Ontario, parents reported more parent level and environmental level barriers to support child PA compared to other behaviors such as reducing recreational screen time, encouraging healthy eating, and ensuring good sleep habits [36]. We therefore propose that more studies on parenting style are needed to identify the relationship between parenting style and PA in children.

Several methodological points and limitations need to be considered when interpreting the results of this study. First, although the accelerometer is a widely used tool to measure PA, it does not provide information on the types of activities being performed or the physical environment in which the physical activities took place. Additional descriptive studies employing direct observational approaches to capture contextual PA and sedentary time information are therefore warranted. Second, the Activity-Related Parenting Practices Scale was used as a measure of parental support in the four previous studies we cited [13,14,15,16] and the validity has been examined [34], whereas the validity of the questions used in our study have not been examined. One point of difference between the Activity-Related Parenting Practices Scale and our questionnaire is that it analyzed logistical data as a whole not as individual questions, whereas we analyzed the results of each question separately for their association with PA and sedentary time of the children. Moreover, a question of a parent watching sports event is needed to pay attention, because parents cannot watch the sport if the child is not playing sport. Third, we cannot detect reasons of the results, because the children were not asked to complete their activity log sheets together with their parent during the measurement period. In the future study, log sheets, GPS, or both, will be needed to identify together with their mother and/or father. Fourth, almost 70% of the parents were female, which may bias the generalizability of our findings on parent-child associations. Our findings may therefore be more relevant to mother–child relationships. Previous studies have reported a similar issue [28,37,38]. The strengths of our study include the use of a sample population of Japanese primary school children and their parents with the data from both parents and children being collected on the same days within an Asian country. The sample size of our study was also larger than that of most earlier studies.

## 5. Conclusions

In conclusion, objectively evaluated MVPA in children correlated positively with maternal MVPA. However, paternal or maternal sedentary time and paternal MVPA did not correlate with sedentary time or MVPA in children. On the other hand, the percentage of MVPA (%MVPA) in children who spent more time with their mothers on weekends was significantly lower than that of children who spent less time (%MVPA, 8.4% vs. 9.3%). Children whose mothers watched their sports events had a significantly higher % MVPA than those whose mothers did not (%MVPA, 8.9% vs. 8.2%). There were no associations between children’s MVPA and paternal support. The findings of this study demonstrate the significance of maternal MVPA and support to identifying determinants of activity behavior will help interventions to increase PA.

## Figures and Tables

**Table 1:** Physical characteristics of the study participants, grouped according to age and sex.

Variables	Mean ± SD
Boys (n = 166)	
Age (years)	9.4 ± 1.6
Body height (cm)	132.6 ± 10.0
Body weight (kg)	29.8 ± 8.0
Body mass index (kg/m^2^)	16.7 ± 2.6
Body mass index z-score	−0.1 ± 1.3
Sedentary time (%)	46.0 ± 6.9
Total moderate-to-vigorous physical activity (%)	9.6 ± 2.5
Ambulatory moderate-to-vigorous physical activity (%)	5.9 ± 1.9
Non-ambulatory moderate-to-vigorous physical activity (%)	3.7 ± 1.1
Girls (n = 202)	
Age (years)	9.4 ± 1.6
Body height (cm)	132.8 ± 11.3
Body weight (kg)	28.7 ± 7.3
Body mass index (kg/m^2^)	16.0 ± 2.0
Body mass index z-score	−0.2 ± 1.1
Sedentary time (%)	47.5 ± 7.6
Total moderate-to-vigorous physical activity (%)	7.4 ± 2.2
Ambulatory moderate-to-vigorous physical activity (%)	4.0 ± 1.4
Non-ambulatory moderate-to-vigorous physical activity (%)	3.4 ± 1.1

**Table 2:** Responses of children’s’ physical activity to parental support.

Questions		Mother	N	Father	N
How much time do you spend with your child on weekdays?	Low	Less than 5 h	182	Less than 1 h	61
	High	More than 5 h	120	More than 1 h	77
How much time do you spend with your child on weekends?	Low	Less than 5 h	30	Less than 5 h	45
	High	More than 5 h	249	More than 5 h	89
How often do you encourage your child to be physically active?	No	Rarely or Occasionally	140		115
	Yes	Often and Always	161		22
Do you watch your child’s sporting events?	No	Rarely or Occasionally	135		70
	Yes	Often and Always	171		65
Do you think that it is important for you to pay attention to sports?	Unimportant	Unimportant	24		18
	Somewhat important	Somewhat important	89		34
	Important	Important and Very important	189		85
Does your family enjoy sports or physical activities as a family recreational activity?	No	Rarely or Occasionally	204		100
	Yes	Often and Always	102		38

**Table 3:** Associations between objectively evaluated physical activity and sedentary time of children and their mothers or fathers.

Children	Mother (n = 321)			Father (n = 123)		
Variable	Coefficient	SE	*p*-Value	Coefficient	SE	*p*-Value
Sedentary time (%)	0.056	0.043	0.200	0.116	0.056	0.175
Total MVPA (%)	0.168	0.043	<0.001	0.118	0.065	0.213
Ambulatory MVPA (%)	0.207	0.060	0.001	0.043	0.059	0.545
Non-Ambulatory MVPA (%)	0.053	0.028	0.062	0.195	0.063	0.090

MVPA, moderate-to-vigorous physical activity. Mixed linear models were used to adjust for parent’s age, child’s age, gender, BMI z-score as fixed effects, and school as a random effect.

**Table 4:** Associations between objectively evaluated children’s total moderate-to-vigorous physical activity and parental support.

Questions		Mother				Father			
		n	Least Square Mean	SE	*p*-Value	n	Least Square Mean	SE	*p*-Value
How much time do you spend with your child on weekdays?	High	302	8.4	0.2	0.314	138	8.4	0.2	0.388
	Low		8.7	0.2			8.1	0.3	
How much time do you spend with your child on weekends?	High	279	8.4	0.2	0.034	134	8.4	0.2	0.516
	Low		9.3	0.4			8.1	0.3	
How often do you encourage your child to be physically active?	Yes	301	8.8	0.2	0.106	137	8.6	0.2	0.2652
	No		8.3	0.2			7.8	0.3	
Do you watch your child’s sporting events?	Yes	306	8.9	0.2	0.008	135	8.5	0.3	0.557
	No		8.2	0.2			8.2	0.2	
Do you think that it is important for you to pay attention to sports?	Unimportant	302	7.8	0.5	0.492	137	7.5	0.5	0.464
	Somewhat important		8.7	0.3			8.6	0.4	
	Important		8.5	0.2			8.3	0.2	
Does your family enjoy sports or physical activities as a family recreational activity?	Yes	306	8.7	0.2	0.533	138	8.7	0.3	0.250
	No		8.5	0.2			8.1	0.2	

Least square mean was adjusted for child’s age, gender, BMI z-score as fixed effects, and school as a random effect.

**Table 5:** Associations between objectively evaluated children’s sedentary time and parental support.

Questions		Mother				Father			
		n	Least Square Mean	SE	*p*-Value	n	Least Square Mean	SE	*p*-Value
How much time do you spend with your child on weekdays?	High	302	47.1	0.7	0.380	138	46.1	0.8	0.663
	Low		46.4	0.6			46.6	0.8	
How much time do you spend with your child on weekends?	High	279	47.0	0.6	0.037	134	46.3	0.7	0.926
	Low		44.4	1.2			46.2	1.0	
How often do you encourage your child to be physically active?	Yes	301	46.7	0.7	0.726	137	45.7	0.7	0.409
	No		46.9	0.7			47.3	0.9	
Do you watch your child’s sporting events?	Yes	306	46.1	0.6	0.055	135	46.6	0.8	0.566
	No		47.6	0.7			45.8	0.8	
Do you think that it is important for you to pay attention to sports?	Unimportant	302	47.0	1.3	0.409	137	46.7	1.6	0.591
	Somewhat important		47.3	0.8			44.9	1.2	
	Important		46.4	0.6			46.9	0.7	
Does your family enjoy sports or physical activities as a family recreational activity?	Yes	306	46.7	0.8	0.950	138	45.6	1.1	0.527
	No		46.7	0.6			46.6	0.7	

Least square mean was adjusted for child’s age, gender, BMI z-score as fixed effects, and school as a random effect.
